# The 4-item family resilience scale: psychometric evaluation and measurement invariance of the malay version in adolescents and young adults

**DOI:** 10.1186/s40359-023-01435-5

**Published:** 2023-11-13

**Authors:** Hazalizah Hamzah, Chee-Seng Tan, Fatanah Ramlee, Syara Shazanna Zulkifli

**Affiliations:** 1 Department of Psychology, Faculty of Human Development, Sultan Idris Education University, Perak, Malaysia; 2 School of Psychology, College of Liberal Arts, Wenzhou-Kean University, Zhejiang, China

**Keywords:** Validation, Family resilience, Adolescents, Young adults, Malaysia, Reliability

## Abstract

**Background:**

The original Family Resilience Scale (FRS) is a reliable tool to assess family resilience. However, the FRS is based on the United States and parental context. Thus, the usefulness of the FRS for the adolescent and young adult population in Asian countries, particularly Malaysia remains unknown. This study translated the FRS into the Malay language and validated it on Malaysian adolescents and young adults to identify its potential as a self-report tool to assess the resilience level of their family.

**Methods:**

A total of 351 participants (*M*_*age*_ = 19.75, *SD*_*age*_ = 3.29) were recruited in the study using purposive sampling. Confirmatory factor analysis was conducted to examine the factorial structure of the Family Resilience Scale-Malay (FRS-Malay) and measurement invariance between adolescents and young adults. Then, the scale’s reliability was investigated using Cronbach’s alpha, McDonald’s omega coefficients, and composite reliability index. Finally, we examined the discriminant validity of the FRS-Malay by correlating its score with individual resilience score and examined the incremental validity of the scale using hierarchical multiple regression analysis to test if family resilience can explain individual well-being levels beyond and above individual resilience.

**Results:**

The findings of the confirmatory factor analysis suggest that a single-factor model is supported for both age groups. Furthermore, the scale exhibited scalar invariance between adolescents and young adults. The scale also exhibited good reliability, as the value of Cronbach’s alpha, McDonald omega coefficients, and composite reliability index were above 0.80. Additionally, the Pearson correlation analysis showed a positive correlation between the FRS-Malay and individual resilience scores, which supports the discriminant validity of the scale. Similarly, the incremental validity of the scale is also supported. Specifically, family resilience had a positive correlation with well-being, even after controlling for individual resilience in the regression analysis.

**Conclusions:**

The FRS-Malay has demonstrated good reliability and validity. The scale measures the same construct of family resilience across adolescents and young adults, making it suitable for comparisons. Therefore, this unidimensional tool is appropriate for self-reporting their perceived level of family resilience. It is also useful for studying the development and fluctuation of family resilience in the Malaysian context.

Adolescents and young adults are two crucial phases that shape an individual’s personality, life goals, and overall well-being. These age groups are at the identity exploration stage and are vulnerable to external and internal influences that mould their future selves. Resilience is a skill that helps young people overcome these negative influences and allow for a better and healthier life in the future.

Resilience is defined as the process of positive adaptation in dealing with adversity, trauma, tragedy, threats, or stressors relating to social relationship issues, physical health problems and financial stressors which may enhance personal growth [1]. In general, resilience has been found to be important for children in their childhood development [2], university students to deal with their stress in academic learning [3], and healthcare providers to cope with their difficult situations in clinical settings [4].

Specifically for adolescents and young adults, resilience equips them with resources and opportunities to face life challenges, and those who possess resilience exhibit favourable relationships with their parents, alongside proficient cognitive, and social-emotional development [5]. Research data from a handful of studies among adolescents and young adults has also identified resilience as a predictor of academic, psychological, and social achievements [6], mental illness [7], and effective coping mechanisms [8].

A related but distinct construct of resilience is family resilience. Family resilience is the extent to which families can successfully overcome life challenges as a family unit and function to benefit individual family members [9]. The level of family resilience determines whether families are disrupted by crises or persistent obstacles, or whether they become more resilient and resourceful [10]. On top of resilience, family resilience is also beneficial to the development of adolescents and young adults. The concept of family resilience encompasses various factors, including parental power, the dynamics within the family unit, interdependence among family members, and the social context within the family [11]. Family resilience provides a valuable framework for identifying and strengthening crucial systems that enable families to withstand crises and challenges by strengthening interactional processes and affirming their potential, thereby empowering families to endure and recover from disruptive circumstances [9].

Family resilience is associated with increased parental well-being [12] and indirectly enhances individual resilience by increasing perceived social support [13]. More recently, studies on family resilience reported improved flourishing among children [14], heightened post-traumatic growth and quality of life [15], weakened the impact of adverse childhood experiences on mental health and behavioural disorders among children [16], and easing the adaptation to abrupt adversities such as the recent COVID-19 pandemic [17].

According to the family resilience framework [18], hardship inside the family has a ripple effect that impacts everybody including the youngsters. This framework denotes that when youngsters participate in family interactions to accomplish a common objective, these interactions help them cultivate social virtues, prosocial behaviours, and a sense of belonging that exceeds the home setting. Family resilience helps to overcome adversity and gain a sense of efficiency, confidence, and health [19].

Although the construct of family resilience is widely used, a review of past studies suggests measures of family resilience were developed in various contexts. A systematic literature review by Zhou and colleagues [20] has outlined several current measures of family resilience, including the Family Resilience Assessment Scale [21], Multidimensional Family Resilience Assessment: The Individual, Family, and Community Resilience Profile [22], Family-Resilience Scale for Veterans [23] and Family Resilience Inventory [24]. Most of the scales comprise many items, ranging between 21 and 75 items and most of the scales are assessed for individuals above 18 years old [20]. Therefore, the systematic review showed no family resilience scale that has few items with good reliability and from the adolescent’s point of view and hence, could not be generalized to the adolescent population [20].

The FRS by Zhang et al. [25] is a measure that overcomes these issues by developing a reliable and valid measurement of family resilience with only 4 items that are direct and succinct. The FRS tops the other family resilience scales in the literature by having only four items with good reliability values (α = 0.89) [21]. Furthermore, the four items within the scale are in line with the family resilience framework brought upon by Walsh [18], focusing on family interactions to build up family resilience. It is important to note that the original FRS is still newly developed, hence, the researchers found no other studies replicating the FRS in other populations. The present study will contribute to the field of resilience by providing a reliable and valid instrument to assess the level of family resilience among adolescents and young adults in the Malaysian context.

Furthermore, adolescents and young adults, also known as Generation Z have been written off as wanting immediate outcomes, impatient, quick-thinking, difficulty sustaining focus, and immature level of knowledge [26, 27]. Therefore, long-form instruments are not practical for them and may result in inaccurate self-reports. In addition, between 2010 and 2017, approximately 14–16% of those aged 15 to 24 years old had no education [28], further justifying the need for a short and simple questionnaire. Moreover, as evident, longer questionnaires increase the non-response rate and reduce the time taken for participants to answer each question [29], indicating that they may not read and understand the items fully. Furthermore, studies have shown that shorter questionnaires tend to achieve better response rates than longer questionnaires [30, 31] with better reliability values [32].

## Family resilience scale – malay version (FRS-malay)

The brief 4-item FRS not only shows good psychometric properties but also has the advantage of reducing the cognitive burden of respondents. Despite the strengths, it is important to note that Zhang and colleagues’ analysis was based on the responses provided by caregivers in the United States rather than their children [21]. As a result, the usefulness of the FRS in other populations (e.g., adolescents) and cultural contexts remains unknown. Although researchers may benefit from using FRS to identify family resilience from the perspectives of the caregivers, identifying family resilience directly from adolescents and young adults is necessary as there may be discrepancies in family resilience between the caregivers’ perspectives with adolescents’ and young adults’ perspectives. For example, the caregiver may perceive that they talk together about what to do and solve problems together, but this may be different from the adolescent’s perspective. The adolescent might feel that they did not talk together and work together as much as the caregiver feels. Hence, the agreeableness of the scale may be different. Therefore, a combination of all three perspectives may be beneficial for researchers to identify the similarities and differences in family resilience levels and aid in future research exploration.

The present study addressed this gap by validating the FRS on adolescents and young adults in Malaysia. Moreover, to minimize the language barriers and misunderstanding of the items, the present study translated the (English version of the) FRS into the Malay language (FRS-Malay). Although Malaysia is one of the most proficient countries in English, the Malay version of this instrument is designed to cater for Malay-speaking adolescents and young adults who are not well-versed in the English language. This is in accordance with a Malaysian study reporting that adolescents and young adults have poor literacy in the English language [33, 34]. The inability to understand the English language makes it difficult for these age groups to complete the brief FRS, and in addition, will require extra effort from the researchers and enumerators to assist in the administration. Furthermore, as English is not the native language of Malaysia, it is natural to translate the instrument to the native language, Malay for wider applicability and easier understanding.

Finally, it is of utmost importance to test the validity of the translated instruments to ensure their reliability and clarity. For example, after translating it into the Malay language, some items of a self-rated creativity scale, which shows good psychometric properties among young adults in the Malay language, have been found not applicable to adolescents [35]. Therefore, investigating the psychometric qualities of the translated version of FRS is critical [36] before administering it to Malaysian adolescents and young adults in other future studies.

Taken together, the present study aimed to shed light on the psychometric properties of the FRS-Malay to understand whether the scale is a potential tool for adolescents and young adults to self-report the resilience level of their families. On top of testing the factorial structure and reliability, convergent and incremental validity were also examined in this study. Finally, if the best-fit model for the two groups is identical, we examined measurement invariance between age groups, which are adolescents and young adults to understand if the scale is invariant between the two groups and could be administered to young adults as well.

## Methods

### Participants

The sample size was determined using various rules-of-thumb provided for confirmatory factor analysis. The total number of items for FRS is only 4 items, while for resilience and well-being are 25 and 5 items, respectively. Hence, using the widely accepted ratio of a minimum of 5 to 10 respondents per indicator [37, 38], this research minimally requires around 170 respondents for each age group. A total of 351 participants (*M*_*age*_ = 19.75, *SD*_*age*_ = 3.29), comprising 169 adolescents, and 182 young adults were recruited in the study using purposive sampling.

Following the World Health Organisation [39], the ages for adolescents are between 10 and 19 years old, and thereupon, the young adults group in this study are classified as those above 19 years old. Participants were included following the inclusion criteria: (a) adolescents and young adults, (b) have basic literacy in the Malay language, and (c) of Malaysian nationality. The questionnaire was circulated through online and offline mediums. They completed either an online survey using Google form or printed surveys consisting of the demographic section, family resilience scale, resilience scale, and WHO well-being scale, all of which were back-to-back translated into the Malay language by the research team.

Prior to answering the questionnaires, on the first page of the questionnaire, participants were briefed on the introduction and purpose of research, research team background information, informed consent, ethical descriptions that their data would be anonymous and confidential, their rights to withdraw from completing the questionnaire at any given time, consent for publication of findings, and an agreement statement that participants give their consent in this study once they completed the questionnaire. Informed consent was obtained by all participants included in this study. The participants were categorized into adolescents aged 13 to 19 years (*n* = 169, *M* = 16.70, *SD* = 1.88) old, and young adults aged 20 to 24 years old (*n* = 182, *M* = 22.59, *SD* = 0.89). Table 1 shows the participants’ demographic background.

**Table 1:** Participants’ demographic background

Demographic Information	*f*	%
Age Group		
Adolescent	169	48.2
Young adults	182	51.8
Gender		
Adolescent		
Male	67	39.6
Female	102	60.4
Young adults		
Male	40	22.0
Female	141	77.5
Did not state	1	0.5
Race		
Malay	286	81.5
Indian	12	3.4
Chinese	19	5.4
Bumiputra	34	9.7
Current Education Level		
No education	2	0.6
Secondary school	125	35.6
Pre-University	28	8.0
Diploma	7	2.0
Bachelor and above	189	53.8
**Total**	**351**	**100.00**

### Procedure

This research was approved by the Human Research Ethics Committee of Sultan Idris Education University (Code: UPSI/PPPI/PYK/ETIKA(M)/014(643)). Following approval from the institution’s ethics committee, the research team distributed the surveys using paper surveys and online (i.e., social media platforms) from November to December of 2022. Post-data collection, the data were examined for any missing values or data input errors. There were no missing values found for this data. The data were then analysed with descriptive statistics and confirmatory factor analysis to evaluate their reliability and validity.

### Measurements

This study utilizes three instruments, namely the Resilience Scale [40], the Family Resilience Scale [21], and the Malay Version of the WHO-5 Well-Being Scale [41].

### Family resilience scale

The family resilience measure developed by Zhang et al. [21] investigates the level of resilience in a family from the participant’s perspective. A total of four questions were asked on a 4-point Likert scale ranging from 1 (All of the time) to 4 (None of the time). The instruction for the instrument is “When your family faces problems, how often are you likely to do each of the following,” and the four items for the original and translated instrument are “Talk together about what to do/*Berbincang tentang perkara yang hendak dilakukan*,” “Work together to solve our problems/*Menyelesaikan masalah bersama-sama*,” “Know we have strengths to draw on/*Menyedari bahawa kami mempunyai kekuatan*,” and “Stay hopeful even in difficult times/*Tidak berputus asa dalam menghadapi kesukaran*.” To calculate the total score for family resilience, each item is reverse-scored and averaged. Higher scores indicate a more resilient family, and vice versa. The scale obtained a good reliability value of α = 0.89 in the original paper, and this study obtained α = 0.83 for both adolescents and young adults. The FRS measured family resilience from the perspective of caregivers. It is important to note that the researchers failed to identify other studies replicating the usage of the FRS. Accordingly, this research adopted and translated the FRS into the Malay language and evaluated its suitability to be administered to Malaysian adolescents and young adults.

### Resilience scale

The resilience scale developed by Wagnild and Young [40] comprises a comprehensive set of 25 statements of two distinct subscales, namely personal competency and acceptance of self and life. The participants responded to the survey using a 7-point Likert scale, ranging from 1 (disagree) to 7 (agree). All items within the scale are positively scored and are aggregated to generate a composite score of resilience. Higher scores on this scale indicate a greater level of resilience. In contrast, lower scores indicate a lower level of resilience when faced with adversities in life. The scale was adapted into the Malay language for research purposes. Examples of items include “When I make plans I follow through with them/*Apabila saya membuat perancangan, saya mengikuti perancangan tersebut*”, and “I can usually look at a situation in a number of ways/*Pada kebanyakan masa, saya dapat melihat sesuatu situasi dari pelbagai sudut*”. The original scale had a good reliability value of α = 0.91. The scale has also been extensively used within Malaysia on samples of adolescents, such as juvenile delinquents [42], adolescent refugees [43], late adolescents [44] and young adults, such as Malaysian undergraduates [45, 46]. The reliability value for the resilience scale in the original paper was α = 0.91, and in this study is α = 0.89 and α = 0.90 for adolescents and young adults, respectively.

### WHO-5 well-being scale

The present study utilises the Malay version of the WHO-5 Well-Being scale, as translated by Suhaimi et al. [41]. The scale originates from the World Health Organisation (WHO) [47] in 1998. The scale comprises a set of five items designed to assess an individual’s level of well-being. Examples of items include “I feel cheerful and in good spirit/*Saya rasa ceria dan bersemangat*” and “I feel calm and relaxed/*Saya rasa tenang dan relaks*”. The participants provided their responses on a 6-point Likert scale, ranging from 0 (none of the time) to 5 (all of the time). The items are formulated in a positive manner and are added together to determine the total score for well-being. Higher scores indicate a better well-being level. The Malay version has a reliability value of α = 0.91. The scale is one of the most widely used instruments to measure well-being, and particularly in Malaysia, the scale has been validated among adolescents [48], young adults [49], adults with diabetes patients [41], and the elderly [50]. In this study, the reliability values of the well-being scale are α = 0.88 and α = 0.92 for adolescents and young adults, respectively.

### Back-to-back translation

The back-to-back translation process follows the suggestions by Sperber [51]. The research team translated the scale from the original language (English) to the target language (Malay). Then, two independent language experts without prior knowledge of the scale back-translated it into the English language for comparison of item meaning and structure, as well as identifying problematic items. The revised scale was evaluated during pilot testing, and the reported a reliability value of α = 0.83. The family resilience scale was paired with the resilience scale (α = 0.90) and well-being scale (α = 0.90) for validation purposes.

### Data analysis

The data analysis was conducted using the statistical software R, version 4.2.0 for Windows, developed by R Core Team [52]. Specifically, we used the lavaan package, version 0.6–11 by Rosseel [53] to carry out confirmatory factor analysis (CFA) and measurement invariance test using weighted least square mean and variance adjusted (WLSMV) estimator to address the ordinal data to examine if the original unidimensional model is fit to the data of adolescents and young adults, respectively. We referred to the following cut-off values to identify a good-fit model: the ratio of chi-square value to degrees of freedom (χ2/df) $\le$ 3, comparative fit index (CFI) and Tucker–Lewis index (TLI) > 0.95, root mean square error of approximation (RMSEA) ≤ 0.06, and in standardized root mean square residual (SRMR) < 0.08 [54]. The abovementioned cut-off values have been widely used in the literature; however, it is noteworthy that those values were generated from a simulation using a maximum likelihood estimator. Their usability remains open when a different estimator is used. Therefore, we mainly referred to the SRMR, which has been found to be robust to different estimators [55], for identifying good-fit models, while the other indicators were reported for reference.

If the best-fit model for the two age groups is identical, a 3-step measurement invariance (MI) test was then conducted to clarify if the FRS-Malay is invariant between the groups. In the first step, we examined the configural invariance to understand if the factor structure is invariant between the groups. Next, the metric invariance was tested to examine if the factor loadings were equivalent in both groups. Finally, the scalar invariance was tested to evaluate if the item thresholds were comparable in both groups. Residual invariance was not tested because a strict invariance is unnecessary and is rarely to be attained in the applied context (e.g., Kline [56]; Little [57]). Considering the robustness of SRMR and following Chen’s [58] recommendation, the assumption for metric invariance is supported in the present study when a change in SRMR (∆SRMR) < 0.030, while the scalar invariance is supported if an ∆SRMR < 0.010. Besides that, we also planned to conduct the Fisher’s Z test to examine the significant differences in the correlation between the two age groups.

The Cronbach’s alpha (α) and McDonald’s omega (ω) coefficients as well as the composite reliability were computed to examine the reliability of the scale. We also computed the composite reliability to further examine the scale. The three indicators were examined using the *reliability* and *compRelSEM* functions of the semTools R-package version 0.5-6 [59] respectively. Finally, the discriminant validity of the FRS-Malay was tested by correlating its score with the individual resilience score. Although family resilience is positively related to individual resilience [13, 60], they are conceptually different. Specifically, family resilience refers to the ability to bounce back from adversities at the family level, while individual resilience focuses on the personal level. Therefore, it is essential to demonstrate that the FRS-Malay is not measuring individual resilience. Meanwhile, the concurrent validity of the scale was tested by examining the relationship between family resilience and well-being. This is because family resilience has been found to benefit individual well-being [15, 61]. Furthermore, we examined the incremental validity of the FRS-Malay using multiple hierarchical regression with individual resilience and family resilience as the predictors and individual well-being as the outcome variable.

## Results

### Confirmatory factor analysis

Table 2 summarizes the CFA results. The hypothetical unidimensional structure of the FRS-Malay was supported for both adolescents and young adults. Inspection of the adolescents? results showed that all indicators are within the suggested range except for the *χ*^*2*^/df (38.43 for adolescents, 9.46 for young adults). The standardized factor loadings for items 1 to 4 were .859, .829, .754, and .547. The results for young adults, however, are mixed. Among the indicators, only CFI and SRMR were within the acceptable range. The factor loadings were .750, .848, .748, and .563 for items 1 to 4.

**Table 2:** Goodness-of-fit indices for tests of invariance of family resilience scale-malay for adolescents and young adults

	*χ* ^*2*^	*df*	CFI	TLI	RMSEA [90% CI]	SRMR	Composite Reliability		Cronbach alpha			McDonald Omega	
Baseline Model													
Adolescent (n = 169)	230.607***	2	0.991	0.974	0.076 [0.000, 0.187]	0.023	0.848		0.834			0.848	
Young Adult (n = 182)	18.924***	2	0.925	0.776	0.216 [0.134, 0.310]	0.074	0.830		0.818			0.829	
**Measurement Invariance**	***χ*** ^***2***^	***df***	**CFI**	**TLI**	**RMSEA** **[90%CI]**	**SRMR**	**Model Comparison**	**∆** *χ* ^***2***^	***df***	***p***	**∆CFI**	**∆RMSEA**	**∆SRMR**
Model 1: Configural invariance	26.845***	4	0.949	0.848	0.181 [0.120, 0.249]	0.041	-	-	-	-	-	-	-
Model 2: Metric invariance	22.142**	7	0.966	0.942	0.111 [0.061, 0.165]	0.044	2 vs. 1	4.703	3	0.195	0.017	0.070	0.003
Model 3: Scalar invariance	23.707**	10	0.970	0.964	0.089 [0.043, 0.135]	0.045	3 vs. 2	1.565	3	0.667	0.004	0.022	0.001

*Note*: The reported indices were based on robust values corrected in accordance with the WLSMV estimator

*χ*^*2*^ = chi-square value, *df* = degrees of freedom, CFI = comparative fit index, TLI = Tucker-Lewis Index, RMSEA = root-mean-square error of approximation, CI = confidence interval, SRMR = standardized root mean square residual, ∆ *χ*^*2*^ = difference in *χ*^*2*^, ∆CFI = difference in CFI, ∆RMSEA = difference in RMSEA, ∆SRMR = difference in SRMR

### Measurement invariance

Based on the SRMR value, the unidimensional model is acceptable for both age groups. Therefore, a measurement invariance test was carried out to investigate if the FRS-Malay is consistent between adolescents and young adults. Despite the configural invariance model showing inconsistent results, we referred to the SRMR value and concluded that the one-factor structure applies to adolescents and young adults (see Table 2). A similar pattern was observed in the metric invariance model. Moreover, the ∆SRMR was < 0.03 indicating that factor loadings are equivalent in both age groups. Finally, all indicators except for RMSEA showed that the scalar invariance model fit the data. More importantly, the ∆SRMR was 0.001 indicating that item thresholds are comparable in both groups.

### Reliability and validity

Table 3 shows the descriptive statistics, reliability, and correlation results for the three measurements used in the present study for the two age groups. The FRS-Malay as well as the resilience scale and WHO-5 showed good internal consistency for the adolescent group. The Cronbach alpha coefficients were greater than 0.833 while the McDonald omega coefficients were above 0.845. Moreover, the composite reliability of the FRS-Malay was 0.848. Similarly, all measurements were found to have good to excellent internal consistency in the young adult group: α ranged from 0.825 to 0.917 and ω ranged from 0.835 to 0.918, while the composite reliability of the FRS-Malay was 0.830.

**Table 3:** Descriptive statistics, reliability, and intercorrelation for adolescents and young adults

No	Variable	Min	Max	Mean	*SD*	Skewness^a^	Kurtosis^b^	Cronbach	Omega	1	2	3
Adolescents (n = 169)												
1	Family Resilience	1	4	2.891	0.738	-0.047	-0.978	0.834	0.846	1		
2	Resilience	25	75	123.840	18.695	-0.457	0.996	0.894	0.896	0.359***	1	
3	Well-Being	0	100	60.828	20.980	0.286	-0.851	0.875	0.873	0.335***	0.588***	1
Young Adults (n = 182)												
1	Family Resilience	1	4	3.125	0.702	-0.264	-0.935	0.825	0.835	1		
2	Resilience	25	75	131.951	17.842	-0.834	1.144	0.898	0.901	0.564***	1	
3	Well-Being	0	100	63.143	22.003	0.043	-0.785	0.917	0.918	0.580***	0.671***	1

*Note*: Min = minimum, Max = Maximum, *SD* = Standard deviation; Cronbach = Cronbach alpha coefficient; Omega = McDonald omega coefficient

*** *p* < .001

^a^Standard error = 0.187 for adolescents, 0.180 for young adults

^b^Standard error = 0.371 for adolescents, 0.358 for young adults

In addition, we also conducted an items analysis. Table 4 shows the descriptive statistics, corrected item-total correlation, Cronbach’s alpha if the item is deleted, and interitem correlation results for the two age groups respectively. For the adolescent group, the corrected item-total correlation coefficients exceeded the suggested cutoff of 0.30 [62], suggesting the items measure the same construct. On the other hand, it was found that removing item 4 can increase Cronbach’s alpha efficiency, though the change is minor. Moreover, the interitem correlation ranged from 0.411 to 0.741. Item 4 was found to have a low correlation with the other items. The same pattern of results was also documented in the young adult group.

**Table 4:** Analysis of the items in the family resilience scale-malay version

	*M*	*SD*	Corrected item-total correlation	Cronbach’s Alpha if item is deleted	Item 1	Item 2	Item 3
Adolescents (n = 169)							
Item 1	2.79	0.94	0.753	0.749	1.000		
Item 2	2.75	0.94	0.727	0.761	0.741	1.000	
Item 3	2.89	0.91	0.681	0.783	0.620	0.622	1.000
Item 4	3.14	0.82	0.504	0.854	0.465	0.411	0.453
Young adults (n = 182)							
Item 1	3.04	0.94	0.653	0.766	1.000		
Item 2	2.99	0.92	0.721	0.731	0.732	1.000	
Item 3	3.13	0.83	0.678	0.755	0.485	0.603	1.000
Item 4	3.34	0.80	0.517	0.824	0.375	0.387	0.588

*Note*: *M* = Mean; *SD* = Standard deviation

The discriminant validity of the FRS-Malay is also supported. Pearson correlation analysis (see Table 3) showed that the FRS-Malay score was moderately and positively correlated with individual resilience score at the 0.001 level among adolescents (*r* = .359) and young adults (*r* = .564), respectively. Likewise, the FRS-Malay also demonstrated good concurrent validity. There was a positive relationship between the FRS-Malay score and individual self-reported well-being among adolescents (*r* = .335) and young adults (*r* = .580), respectively.

On an exploratory basis, we conducted the Fisher’s Z test using an online calculator provided by Lenhard and Lenhard [63] to examine if the differences in the correlation between the two age groups are statistically significant. Results showed that the relationship between the FRS-Malay score and individual resilience was stronger in young adults than adolescents (*Z* = 2.55, *p* = .005). A similar pattern was also observed in the relationship between the FRS-Malay score and well-being (*Z* = 2.956, *p* = .002).

In addition, a hierarchical multiple linear regression (individual resilience was entered in Step 1, while family resilience was entered in Step 2) was conducted to clarify if the relationship between family resilience and individual well-being is confounded by the correlation between individual resilience and well-being. After statistically controlling for the positive relationship between individual resilience and well-being, family resilience was found to have a positive association with well-being in adolescents, (unstandardized coefficient) *B* = 4.048, *p* = .033 (see Table 5 for details). The model explained 35.60% of the total variance (change in R^2^ [Δ*R*^2^ ] = 0.014). A similar pattern was observed in young adults (*B* = 9.246, *p* < .001) and the model explained 50.40% of the total variance (Δ*R*^2^ = 0.056).

**Table 5:** Hierarchical multiple regression analysis summary for individual resilience and family resilience predicting well-being

Adolescents (n = 169)										
	Step 1					Step 2				
Variable	B	95% CI		*SE*	β	B	95% CI		*SE*	β
Constant	-19.724	-36.851	-2.598	8.675		-24.427	-41.916	-6.938	8.858	
Individual resilience	0.650***	0.514	0.787	0.069	0.588	0.594***	0.449	0.739	0.073	0.537
Family resilience						4.048*	0.324	7.773	1.886	0.142
Adj. *R*^*2*^	0.342					0.356				
F	*F*(1, 167) = 88.238, *p* < .001					* F*(1, 166) = 4.605, *p* = .033				
**Young Adults (n = 182)**										
Constant	-46.094	-63.993	-28.195	9.071		-47.935	-64.907	-30.964	8.600	
Individual resilience	0.828***	0.693	0.962	0.068	0.671	0.623***	0.469	0.777	0.078	0.505
Family resilience						9.246***	5.325	13.167	1.987	0.295
Adj. *R*^*2*^	0.448					0.504				
F	*F*(1, 180) = 147.664, *p* < .001					* F*(1, 179) = 21.651, *p* < .001				

*Note*: B = Unstandardized regression coefficient; CI = Confidence interval; SE = standard error; β = Standardized regression coefficient; Adj. *R*^*2*^ = Adjusted *R*^*2*^ value

*p < .05; ** *p* < .01; *** *p* < .001

## Discussion

The present study translated the 4-item FRS [21] into Malay language and validated it on adolescents and young adults in Malaysia. Results showed satisfactory psychometric qualities for the translated scale. To the best of our knowledge, this study is the first to translate a Malay version of the FRS and assess its effectiveness on adolescents and young adults. The results of the confirmatory factor analysis indicate that the unidimensional structure is appropriate for both age groups. Moreover, the results of the measurement invariance test lend support to the scalar invariance of the FRS-Malay, suggesting that the structure, factor loadings, and thresholds are equivalent between the two age groups. Therefore, there is evidence to suggest that the FRS-Malay measures the same constructs in adolescents and young adults, making it possible to compare the family resilience level measured by the scale between the two age groups.

Consistent with the findings of Zhang and colleagues [21] which found good reliability for the family resilience instrument among the caregivers, the FRS-Malay was found to have good reliability among adolescents and young adults. The three indicators used in the present study (i.e., Cronbach alpha, McDonald omega, and composite reliability) consistently showed a value greater than 0.80. The same pattern of results is also observed in young adults. Moreover, the corrected item-total correlation coefficients were greater than 0.50. The findings indicate that the four items measure the same construct and apply to both age groups. The FRS-Malay also demonstrates good validity in the two populations. Supporting the discriminant validity of the FRS-Malay, we found a positive and moderate relationship between family resilience and individual resilience, suggesting that adolescents and young adults who come from resilient families are likely to rate themselves as resilient. The result is not only in line with the literature [13, 61] but also indicates that family resilience is related to but conceptually different from individual resilience.

Notably, the reliability and validity values showed support that the 4-item FRS-Malay reflect the components of family resilience introduced in the family resilience framework [18]. Particularly, each item measures a key process of the framework, including the belief system and communication/problem-solving process. For instance, item 3: ‘Know we have strength to draw on’ focuses on the positive outlook component of the family belief system, while item 4: ‘Stay hopeful even in difficult times’ focuses on making meaning of the adversity component of the belief system, whereby families hold the belief that the crisis is meaningful, manageable, and they have an optimistic view on the outcome of the crisis [18]. Item 1 and 2, respectively, touch upon the communication and problem-solving key processes of the family resilience framework. Item 1: ‘Talk together about what to do’ focuses on the clarity of the crisis at hand to ensure that the meaning of crisis is clear and not ambiguous, while Item 2: ‘Work together to solve our problems’ touches on the collaborative problem-solving element in the key process, whereby families work together by brainstorming or finding opportunities to ensure a shared decision-making process that is fair and able to resolve the crisis at hand [18]. Through this shared belief, families find significance in adversity which promotes a positive outlook on life and encourages better ways for resolving issues, recovery, and development [18].

Our study also found that young adults have stronger relationships between family resilience and individual resilience, and stronger relationships between family resilience and well-being than adolescents, indicating that the impact of family resilience is larger for young adults than adolescents. Quite similar to our findings, a past study has emphasized the importance of family to enhance resilience and was stronger for adults when compared to adolescents [64].

Young adults in this study are categorized as those above 19 years old, and commonly in Malaysia, this age group are in the phase of just graduating from high school, and entering tertiary education. Hence, different challenges were posed for them when compared to the adolescents who are still in school. More autonomy was given to these maturing young adults to direct their future paths such as transitioning to independent living [65], and in return, they require more connections with their family members to stay strong and supported, which helps them to become resilient. Besides that, those who have good relationships with their parents relate closely to their parents when perceiving and approaching problems [66], and hence, their resilience and well-being may be largely influenced by these relations.

Contradictory, adolescents spend a lot of time in school and with their friends, and relationships with peers often extend beyond school hours and compound. This in return strengthened their relationships and contributed the most to resilience building [67]. The limited connections with parents and other family members then contributed to the lower influence of family resilience on individual resilience and well-being.

This is also true in light of the family resilience framework, whereby family resilience is stronger in the presence of shared common goals [18]. Young adults are currently working on securing long-term employment, being independent, sustaining romantic relationships, and obtaining financial security [65]. Young adults, in this sense, have more things in common with their parents than adolescents who have different priorities. Therefore, young adults from resilient families will instil family resilience within themselves, increasing their individual resilience as well as well-being due to being supported by their families as well.

In addition, when the effect of individual resilience was controlled, family resilience continued to have a positive relationship with individual well-being, implying that family resilience can explain one’s well-being beyond individual resilience. The results not only replicate past findings of the beneficial role of family resilience in well-being [15, 61] but also lend support to the incremental validity of the FRS-Malay. In other words, while its role in well-being is modest, especially for adolescents, family resilience is a potential direction to further enhance the well-being of adolescents and young adults and hence, the importance of family shall not be underestimated.

The findings of the present study are useful to family resilience research in three ways. First, our findings translated and validated a measurement of family resilience for Malay-speaking adolescents and young adults. Researchers can now use the scale to collect data for family resilience for cross-cultural studies. The scale is also helpful for local researchers who are interested in family resilience. Specifically, the invariance of the FRS-Malay between adolescents and young adults allows researchers to compare family resilience measured by FRS-Malay between the two populations. In the same vein, researchers can use the FRS-Malay to conduct a longitudinal study and follow the participants from adolescence to young adulthood to investigate the impact of family resilience on the development of individuals. Finally, the comparable psychometric qualities of the English and Malay versions of the FRS suggest that the family resilience concept measured by the scale applies to both Western and Eastern contexts.

While the present study shows promising findings, several limitations deserve attention. First, although the SRMR value suggests that the unidimensional model is acceptable for both age groups, other fit indices (e.g., TLI, RMSEA) exceeded the recommended cut-off range. The inconsistency could be due to the fact that the current items of the FRS-Malay are insufficient to capture adolescents’ and young adults’ perceptions of resilience. For example, item 4 was found to have a relatively low correlation with the other item, suggesting that our participants perceived that somewhat differently from the other items. Researchers may consider using a qualitative approach to explore how different age groups conceptualize resilience. This exploration could lead to the generation of supplementary items, along with the elimination of irrelevant ones, thereby enhancing the overall quality of the scale.

Second, the use of cross-sectional design has limited the scope of the investigation and further examinations are required. For instance, future studies shall examine test-retest reliability to understand the stability of the scale. Likewise, it is essential to examine the predictive validity of the scale to further verify the usefulness of the scale. Besides that, this study uses resilience and well-being as related constructs to family resilience. Other related constructs such as family support, family functioning, and family adjustment which may be better related to family resilience can be used in future studies to evaluate the discriminant validity. Additionally, we did not assess criterion-related validity in this study, which is another limitation that we failed to overcome. Future studies may conduct a rigorous analysis of the validity of FRS-Malay including criterion-related validity to further evaluate the soundness of the instrument.

Third, the present study is unable to examine the measurement invariance between genders due to the small and unequal sample size. As a result, it remains unknown if the FRS-Malay measures the same construct in male and female adolescents and young adults. Researchers are suggested to recruit a representative sample with a balanced number of male and female participants to replicate the present study and examine the invariance between genders. Similarly, this research could not balance the ethnic composition, since the samples are majority from Malay ethnic. Hence, the findings of this study could not be representative of every adolescent and young adult in Malaysia. For better representativeness, future research is encouraged to provide sufficient sample sizes for each majority ethnicity in Malaysia, which are Malay, Indian, and Chinese. In the same vein, replicating the present study on other populations such as children and working adults may further shed light on the usefulness of the FRS-Malay in the Malaysian context.

Finally, although the FRS-Malay shows satisfactory psychometric properties, it is inadequate to generalize the findings to the English-speaking Malaysians. Note that some items of the (original English version) measurements that have been well validated in other cultural contexts have been found to be problematic in the Malaysian context (see Tan, Ong, et al. [68] and Tan, Tee, et al. [69] for example). Future studies, therefore, are warranted to validate the FRS to provide a tool for the English-speaking populations in Malaysia to measure family resilience.

## Conclusion

After analysing the responses obtained from adolescents and young adults in Malaysia, it was found that the Malay version of the 4-item Family Resilience Scale is a useful measurement tool with a unidimensional structure. The scale measures the same construct and can be used to compare family resilience levels between adolescents and young adults. Additionally, family resilience was positively associated with individual resilience. In conclusion, the FRS-Malay is a brief and psychometrically sound measurement tool for family resilience research in Malaysia.

## Data Availability

The datasets used and/or analysed during the current study are available from the corresponding author upon reasonable request.

## Abbreviations

FRS: Family Resilience Scale
FRS-Malay: Family Resilience Scale- Malay
CFA: Confirmatory factor analysis
WLSMV: Diagonally weighted least squares
CFI: Comparative fit index
TLI: Tucker-Lewis index
RMSEA: Root mean square error of approximation
SRMR: Standardized root mean square residual
MI: Measurement invariance

## Declarations

### Disclaimer of using Artificial Intelligence in Writing

The authors declared the use of artificial intelligence (AI), ChatGPT by OpenAI to help polish the writing process of the manuscript in the discussion section, paragraph 9, of the two last sentences to ensure easier understanding of the writings by the readers. The authors fully acknowledged the use of AI and responsibility for the respective content.
